# Genome‐Wide Association Study of Latent Cognitive Measures in Adolescence: Genetic Overlap With Intelligence and Education

**DOI:** 10.1111/mbe.12198

**Published:** 2019-06-30

**Authors:** Georgina Donati, Iroise Dumontheil, Emma L. Meaburn

**Affiliations:** ^1^ Centre for Brain & Cognitive Development Birkbeck, University of London

## Abstract

Individual differences in executive functions (EF) are heritable and predictive of academic attainment (AA). However, little is known about genetic contributions to EFs or their genetic relationship with AA and intelligence. We conducted genome‐wide association analyses for processing speed (PS) and the latent EF measures of working memory (WM) and inhibitory control (IC) in 4,611 adolescents from the Avon Longitudinal Study of Parents and Children. While no loci reached genome‐wide significance, common genetic variants explained 30% of the variance in WM and 19% in PS. In contrast, we failed to find common genetic contributions to IC. Finally, we examined shared genetic effects between EFs and general intelligence, AA and ADHD. We identified significant genetic correlations between WM, intelligence, and AA. A more specific pattern was observed for PS, with modest genetic overlap with intelligence. Together these findings highlight diversity in the genetic contributions to specific cognitive functions and their genetic relationship with educational and psychiatric outcomes.

Executive functions (EF) are cognitive processes controlling thoughts and actions; they allow us to pay attention, resist impulses and change our course of action. Although the structure of EF remains a topic of debate, including potential developmental changes in EF structure (Friedman & Miyake, 2017; Lee, Bull, & Ho, 2013), three core EFs have been more widely examined than others. These are: working memory (WM; holding and manipulating information in mind), inhibitory control (IC; resisting temptations, impulses, and interference) and cognitive flexibility or “shifting” (adapting responses and thoughts to changing demands) (Diamond, 2013; Friedman et al., 2008).

EFs are predictive of academic achievement (AA)—independent of IQ—when measured both using individual tasks and latent factors (Alloway & Alloway, 2010; Cragg & Gilmore, 2014; Rhodes et al., 2016). Numerous cross‐sectional studies show that WM and IC account for significant unique variance in arithmetic, beyond variance explained by IQ, age, processing speed (PS) or reading, in a wide range of age groups (e.g., Bull & Scerif, 2001; Monette, Bigras, & Guay, 2011; see Cragg & Gilmore, 2014 for a review). Associations with WM tend to be larger than with IC or shifting (Cragg, Keeble, Richardson, Roome, & Gilmore, 2017; Friso‐van den Bos, van der Ven, Kroesbergen, & van Luit, 2013), which often do not show unique contributions beyond WM (Cragg et al., 2017; Van der Ven, Kroesbergen, Boom, & Leseman, 2012). WM is also reliably associated with reading, whereas evidence for IC is more varied (Blair & Razza, 2007; Bull & Scerif, 2001; Espy et al., 2004; St Clair‐Thompson & Gathercole, 2006). The finding that IC may not explain any unique cognitive variance over and above that explained by the common variance shared between EFs (“Common EF”) (Friedman et al., 2008) may explain inconsistent results in predicting AA. The associations between EF and AA have been replicated longitudinally (Alloway & Alloway, 2010; Dumontheil & Klingberg, 2012; Mazzocco & Kover, 2007) and across cultures (Lan, Legare, Ponitz, Li, & Morrison, 2011).

PS, although not an EF, has been modeled by many researchers interested in EFs as an important, but separate, cognitive factor due to its key role in supporting and moderating cognition (e.g., Huizinga, Dolan, & van der Molen, 2006; Kail, 2000; see Lee et al., 2013 for discussion). PS is highly correlated with white matter integrity (Kievit et al., 2016) and explains significant unique variance in English and maths attainment during adolescence (Donati, Meaburn, & Dumontheil, 2019; Rohde & Thompson, 2007).

Little is currently known about the genetic architecture of EFs and the extent of shared genetic influence underpinning the observed relationships between EFs and academic achievement. Twin studies have demonstrated that individual differences in EFs are highly heritable, with estimates for latent EF factors ranging from 76% (cognitive flexibility) to 100% (WM), with lower estimates reported for individual EF tasks (29%–76%) (Friedman et al., 2008). Common EF latent measures have reported twin heritability estimates of approximately 99% (Engelhardt, Briley, Mann, Harden, & Tucker‐Drob, 2015; Friedman et al., 2008; OATS Research Team et al., 2012).

Large‐scale genotyping approaches such as genome‐wide association studies (GWAS) seek to identify specific genetic variants (typically, single nucleotide polymorphisms; SNPs) that contribute to the heritability of cognitive abilities in order to identify the associated biological pathways and functions, and gain mechanistic insights (Visscher, Brown, McCarthy, & Yang, 2012). To date, the majority of well‐powered GWASes of cognitive abilities have focused on intelligence (or general cognitive function; *g*); the largest GWAS meta‐analysis of *g* (*N* = 78,308) reported 18 independent SNPs and 30 gene‐based associations (Sniekers et al., 2017)**.** There exist few well‐powered GWASes of specific cognitive abilities; the largest examined individual measures of PS, verbal‐numerical reasoning and short‐term memory (holding information in mind) using the U.K. Biobank sample of adults (*N* = 112,151). It identified two significant SNP associations with PS, but none for short‐term memory (Davies et al., 2016). A smaller study of adults using established single measures of EF such as the trail‐making (*N* = 5,429–6,210) and Stroop tasks (*N* = 12,866) failed to find specific SNP associations with EF, but found an association with PS (*N* = 32,070) (Ibrahim‐Verbaas et al., 2016).

Recently developed statistical approaches use summary statistics generated from GWASes to calculate heritability estimates (*h* ^2^_SNP_) and correlations (*r*_G_) directly from DNA, even when no genome‐wide significant associations have been identified (Bulik‐Sullivan et al., 2015; Dudbridge, 2016). These DNA‐based methods have provided further insights into the extent of shared genetic mechanisms between correlated traits, and the relative contribution of common variants to twin‐based heritability estimates. DNA‐based heritability methods have been used to demonstrate that *g* has a moderate *h* ^2^_SNP_ of between 20% and 35% (Kirkpatrick, McGue, Iacono, Miller, & Basu, 2013; Sniekers et al., 2017); this means that between 20% and 35% of the phenotypic variance in general cognitive ability in the measured population can be explained by the additive effects of all common genetic variants captured in the GWA study. The Davies et al. (2016) GWAS of cognitive functions reported lower estimates for memory (*h* ^2^_SNP_ = 5%) and PS (*h* ^2^_SNP_ = 11%), although this might in‐part be due to the relatively coarse measures used and their low test–retest reliability. Another (smaller) GWAS obtained higher estimates for WM using the *N*‐back task (*h* ^2^_SNP_ = 24%–41%) (Vogler et al., 2014). As expected, large genetic correlations (*r*_G_ ∼.70) have been reported between general cognitive ability and educational attainment (Hill et al., 2018), which indicates that a high proportion of the DNA sequence variants affecting *g* also have an effect on years spent in education (either directly or by mediation). Assessing the extent of generality and specificity of shared genetic influence between cognitive domains and educational outcomes has important implications for understanding why some children do better at school than others, and paves the way for studies centered on understanding causal relationships across development.

The goal of the present study was to bridge the gap in our current understanding of the genetic contributions to specific cognitive abilities in adolescence—a critical developmental period—and their genetic relationship to other educationally relevant measures. To address these aims, three univariate GWASes were performed on latent measures obtained from a principal component analysis (PCA) of cognitive task data collected when participants from the Avon Longitudinal Study of Parents and Children (ALSPAC) were between 9 and 20 years of age (*N* = 4,611); namely (a) WM, (b) IC, (c) and PS (Donati et al., 2019). Linkage disequilibrium score (LDSC) regression was applied to the GWAS summary statistics to (a) estimate the SNP heritability (*h* ^2^_SNP_) of each cognitive measure and (b) index genetic correlations (*r*_G_) between the cognitive measures and between these measures and publicly available GWAS data sets for general intelligence, years in education, and college completion as well as attention deficit hyperactivity disorder (ADHD). We include ADHD due to the large body of research linking it with EF deficits and poorer educational outcomes (Biederman et al., 2004; Clark, Prior, & Kinsella, 2002).

## METHODS

### Study Cohort

ALSPAC (http://www.bristol.ac.uk/alspac/) is an ongoing population‐based study investigating factors influencing development and health. Initial recruitment included 14,541 mothers with 13,988 children alive at age 1. Another round of recruitment at around age 7 left the total sample size for data collected after this age at 15,247 (see Appendix S1 in the online Supporting Information and Boyd et al., 2013; Fraser et al., 2013). The sample for this study consisted of 4,611 participants (2,173 males) aged between 9 years 10 months and 20 years 0 months at testing for whom genome‐wide SNP genotyping data was available. Ethical approval for the study was obtained from the ALSPAC Ethics and Law Committee and the Local Research Ethics Committee.

### Measures

#### *Cognitive Measures*

The three latent cognitive measures used (WM, IC, and PS) were previously derived from a set of 10 cognitive tasks available in the ALSPAC data set during adolescence, broadly defined as between the ages of 10 and 17 years of age. PCA was used as different tasks had been used at different ages, and resulted in a three‐factor solution (see Donati et al., 2019 for full details, and Table S1, Supporting Information for PCA results). Briefly, nine cognitive tasks were included in the three components. The *Counting Span task* (Case, Kurland, & Goldberg, 1982) performed at 10 years of age, is a WM task which requires processing, storing, as well as an element of updating information in WM. The *Stop Signal task* (Logan & Cowan, 1984), performed at 10 and 15 years of age, is a computerized measure of motor response inhibition. Three attention tasks from the Tests of Everyday Attention for Children (adapted from Robertson, Ward, Ridgeway, & Nimmo‐Smith, 1996) were performed at age 11: the *Sky Search task*, assessing selective attention and motor control; the *Dual task*, assessing divided attention; and the *Opposite Worlds task*, which is a shifting task. At age 13 participants were assessed on the *Digit Vigilance task,* which measures sustained attention, and is part of the Cognitive Drug Research computerized cognitive assessment system (Simpson, Surmon, Wesnes, & Wilcock, 1991). *Simple* and *Choice Reaction Time* (RT) measures were also taken at age 13. Finally, a *visuospatial N‐back task* was used at 17 years to test WM and updating. The working memory component of the PCA comprised measures of performance on the *N*‐back, Digit Vigilance, Counting Span, and Dual tasks. The inhibitory control component comprised measures of performance on the Stop Signal task. Finally, the PS component comprised RT measures of the Digit Vigilance, Simple RT, Choice RT, Sky Search, and Stop Signal tasks. No shifting component was identified.

#### *LDhub Measures*

LDhub (http://ldsc.broadinstitute.org/ldhub/) stores the summary statistics from published GWASes. For the purpose of the present study we selected five educationally relevant traits of lifespan intelligence, child intelligence, years spent in education, college completion, and ADHD. The study on lifespan intelligence included 78,308 individuals from 13 different cohorts of European descent: eight cohorts of children <18 years (*N* = 19,509), and five cohorts of adults 18 –78 years (*N* = 58,799). The measure of intelligence for these cohorts was either *g* or a primary measure of fluid intelligence (Sniekers et al., 2017). The study on childhood intelligence includes a child‐only subsample (6–18 years) of 17,989 individuals (*N* discovery = 12,441) also included in the Sniekers et al. study, again using a combination of *g* and fluid intelligence measures (Benyamin et al., 2014).

Years in education is derived from the 2016 Social Science Genetic Association Consortium (SSGAC) GWAS that included adults (<30 years) of European descent (*N* discovery = 293,723; *N* replication = 405,072). The measure of educational attainment used in this study was number of years spent in education (Okbay et al., 2016). The study on college completion included a simpler binary phenotype of whether an individual went to college (university) or not. The replication study included 126,559 individuals (*N* discovery = 101,069) from 42 different cohorts of Caucasian ancestry <30 years (Rietveld et al., 2013). Finally, the study on ADHD (*N* cases = 20,183, *N* controls = 35,191) included 12 cohorts from Europe, North America, and China. Cases had a clinical diagnosis of ADHD (ADHD Working Group of the Psychiatric Genomics Consortium (PGC) et al., 2019).

### Genotyping and Quality Control

Genotyping and imputation was performed by ALSPAC. Adolescents from ALSPAC were genotyped using the Illumina HumanHap550 quad chip by 23andMe subcontracting the Wellcome Trust (Wellcome Sanger Institute, Cambridge, UK) and the Laboratory Corporation of America (Burlington, NC, US). The raw genome‐wide data was subjected to standard quality control procedures to identify individuals and SNPs for exclusion. Samples that passed quality control stages were phased and imputed against the Haplotype Reference Consortium panel using Impute V3 (Delaneau, Marchini, & Zagury, 2012), and post‐imputation SNP and sample quality control was repeated (for full details see Table S2 in the online Supporting Information)**.** The final sample consisted of 4,611 unrelated individuals for whom both cognitive and genotype data were available.

### Statistical Analyses

All data preparation was performed using R (R Core Team, 2013). Prior to analysis all three measures were quantile‐normalized using SNPTEST (Marchini, Howie, Myers, McVean, & Donnelly, 2007) and regressed on age, sex, and the first 10 ancestry principal components. Univariate linear regressions were performed for each of the three measures using SNPTEST v.2 (Marchini et al., 2007). Imputation probability scores were used to maximize statistical power to detect genetic associations. Gene‐based association analyses were performed using MAGMA within the FUMA program using the summary statistics from each GWA analysis (de Leeuw, Mooij, Heskes, & Posthuma, 2015; Watanabe, Taskesen, van Bochoven, & Posthuma, 2017). The proportion of variance attributable to common SNPs (*h* ^2^_SNP_) and genetic correlations (*r*_G_) were estimated from GWAS summary statistics using LDSC regression and cross‐trait LDSC regression as implemented in LDhub (Zheng et al., 2017).

## Results

### SNP Heritability

SNP heritability was 0.30 for working memory, and 0.19 for processing speed (Table 1). The inhibitory control GWAS failed to detect SNP heritability (−0.01) and had a mean *χ* ^2^ < 1, which can be interpreted as a lack of polygenic signal. IC was therefore excluded from further analysis.

**Table 1:** Estimates of SNP Heritability and Phenotypic and Genetic Correlations Between the Three Cognitive Measures

		Phenotypic correlations (R)		
	*h* ^2^_SNP_ (SE)	WM	IC	PS
Genetic correlations *r*_G_ (SE)	WM	**.30 (.07)**	−.03	.13***
	IC	NA	**−.01 (.07)**	.21***
	PS	.24 (0.32)	NA	**.19 (.07)**

*Note*. SNP heritability estimates (h^2^_SNP_) is shown along the diagonal in bold. Phenotypic correlations (*R*) are shown above the diagonal, and genetic correlations (*r*_G_) below the diagonal. IC, inhibitory control; PS, processing speed; SE, standard error; SNP, single nucleotide polymorphism; WM, working memory; NA, genotypic correlations were not calculated for inhibitory control as no SNP heritability was observed for this measure.

*** *p* < .001.

### Genome‐Wide Association Analyses

**Figure 1:**
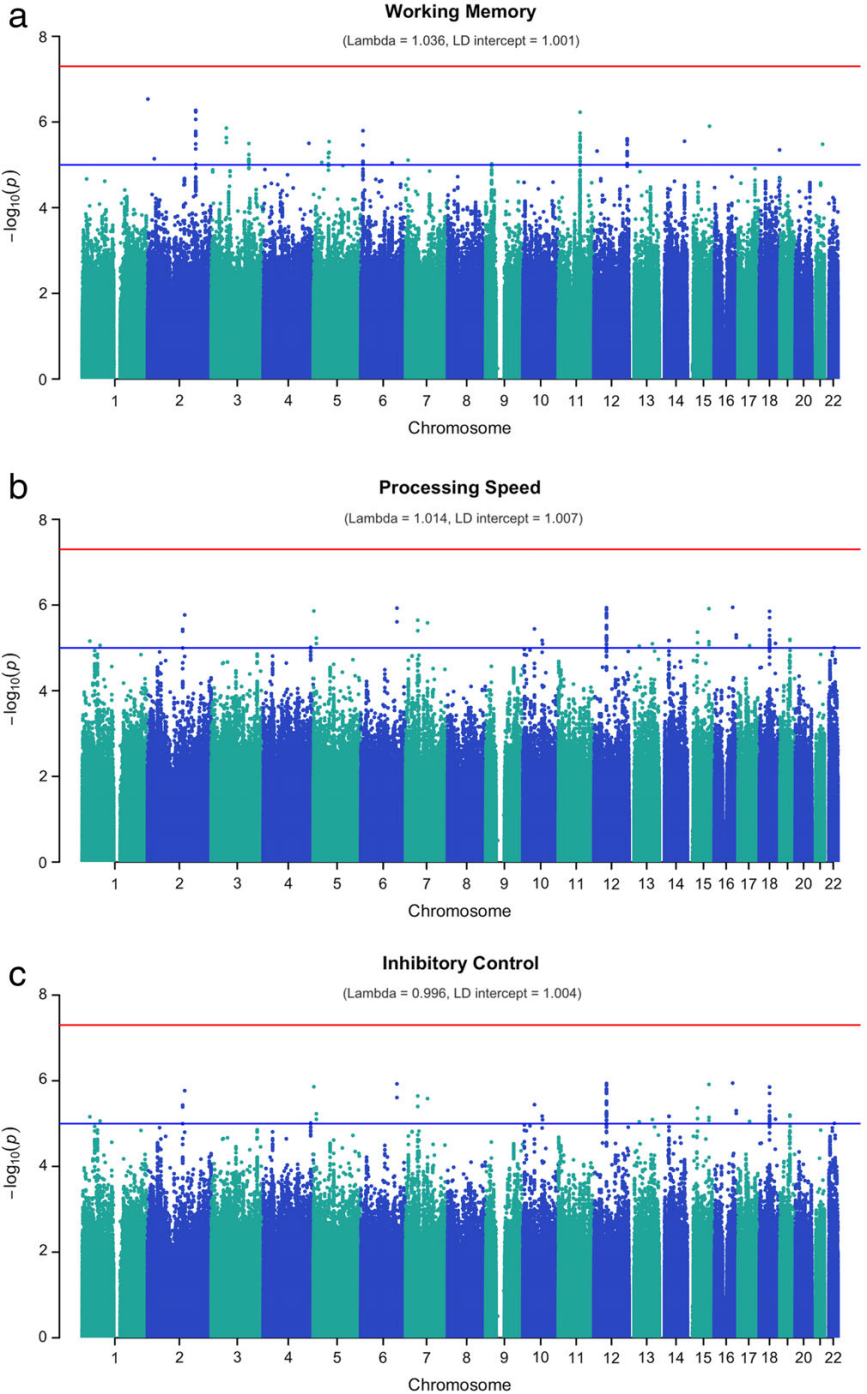
Manhattan plots for GWASes of working memory, inhibitory control, and processing speed. The red dotted line represents genome‐wide significance (*p* < 5 × 10^−8^) and the blue dotted line represents genome‐wide suggestive significance (*p* < 10^−6^).

**Figure 2:**
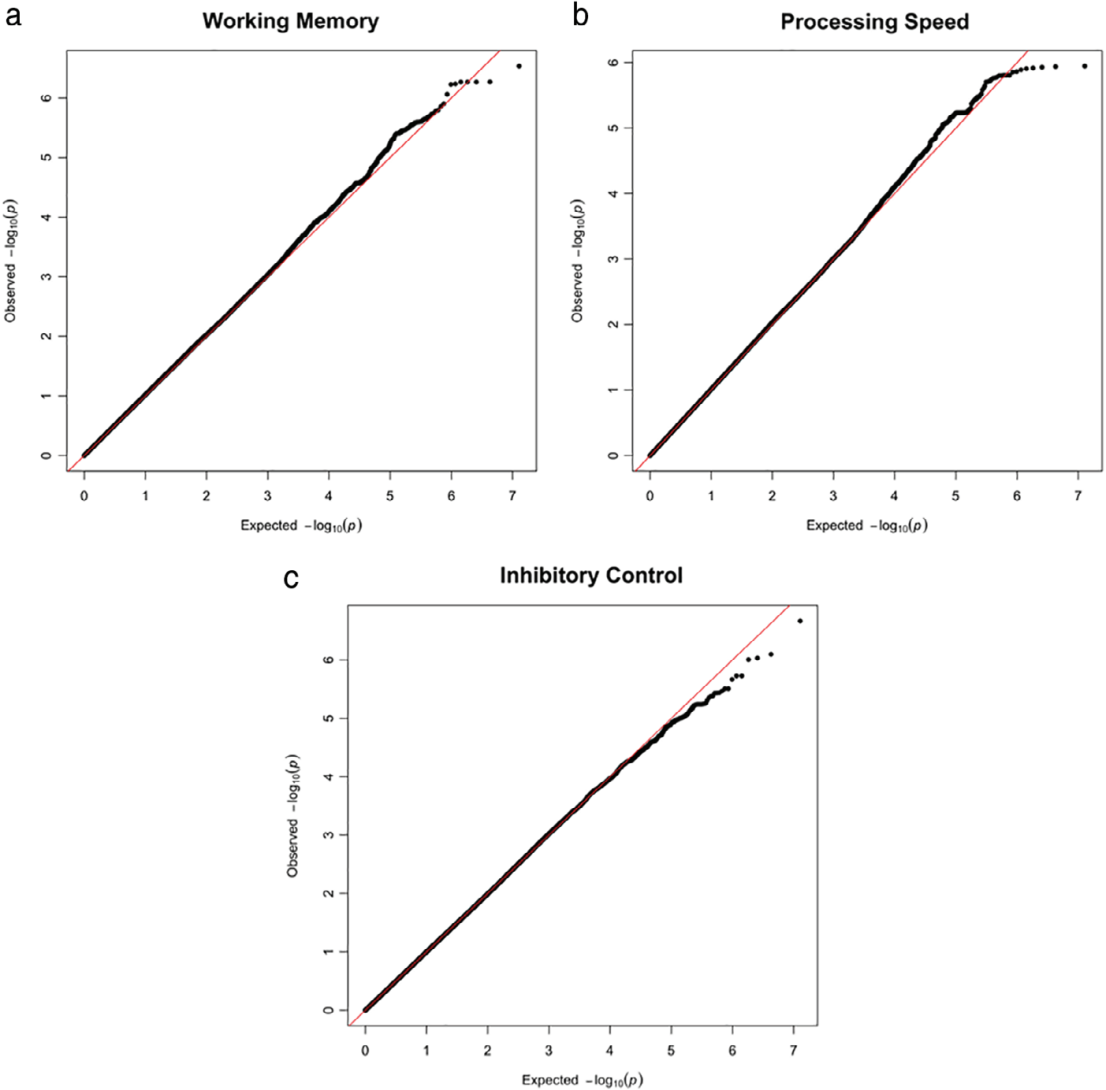
Quantile‐quantile plots (Q‐Q plots) for working memory, inhibitory control, and processing speed GWASes. Q‐Q plots show the distribution of *p*‐values against the expected *p* values. (a) Working memory, (b) Processing speed, (c) Inhibitory control.

Univariate genome‐wide association analyses on the latent measures of working memory, inhibitory control, and processing speed failed to identify any genome‐wide significant SNP associations (*p* < 5 × 10^−8^; Figures 1 and 2). Notably, many of the suggestive SNPs (*p* < 1 × 10^−6^) were located in or close to genes previously reported to be associated with neurocognitive decline, psychiatric disorders, and/or educational attainment (Table S3, online Supporting Information).

### Genetic Correlations Between Cognitive and Related Phenotypes

Genetic correlations were estimated between WM and PS, and with five education‐related phenotypes using the LDhub database (Table 2). Genetic correlations assess the extent to which the same allele (of a bi‐allelic SNP) influences both trait 1 and trait 2. An allele can be associated with a higher or lower score on a measure; thus the direction of the genetic correlation indicates whether alleles act on both traits in the same (positive correlation) or opposite (negative correlation) directions. Therefore, both positive and negative correlations indicate shared genetic etiology. WM was significantly (*p* < .001) genetically correlated with all five phenotypes, with the lowest overlap found for ADHD (*r*_G_ = −.54) which was also the only negative correlation. Genetic correlations for PS were 20%–55% lower than that seen for WM, with nominally significant (*p* < .05) correlations found only for lifespan intelligence (*r*_G_ = .53) and ADHD (*r*_G_ = −.30).

**Table 2:** Genetic Correlations (*r*_G_) With Educationally Relevant Measures Estimated Using GWAS Summary Statistics and LDSC Regression in LD Hub

	Working memory		Processing speed	
	*r*_G_ (SE)	*p* Value	*r*_G_ (SE)	*p* Value
Lifespan intelligence	**1.15 (0.22)**	**1.36 × 10** ^**−7**^	**0.53 (0.27)**	**0.050**
Childhood intelligence	**0.80 (0.16)**	**2.50 × 10** ^**−7**^	0.45 (0.36)	0.211
Years of schooling 2016	**0.70 (0.15)**	**4.20 × 10** ^**−6**^	0.16 (0.16)	0.293
College completion	**0.65 (0.13)**	**5.96 × 10–** ^**7**^	0.19 (0.22)	0.385
Attention deficit hyperactivity disorder	**−0.54 (0.12)**	**1.1 × 10–** ^**5**^	**−0.30 (0.15) **	**0.042**

*Note*. SNP, single nucleotide polymorphism. Bolded figures represent a significant (*p* ≤ .05) correlation. However, Bonferroni correction for multiple testing requires *p* < .001. The genetic correlations can exceed 1 as LD score regression is not a bounded estimator and so can produce correlations larger than 1 or smaller than −1.

### Gene‐Based Analyses

Gene‐based analysis using MAGMA identified two genome‐wide significant associations on chromosome 11: *FAM181B* was associated with WM (*p* = 1.99 × 10^−6^) and *TNNI2* was associated with PS (*p* = 2.74 × 10^−6^; Table 3). *FAM181B* is widely expressed in the brain, and *TNNI2* in the musculoskeletal system.

**Table 3:** Significant Gene‐Based Associations

Phenotype	Chromosome	Gene	Gene name	*p* Value	*z* Statistic
Working memory	11q14.1	ENSG00000182103	FAM181B	1.99 x 10^−6^	4.6124
Processing speed	11p15.5	ENSG00000130598	TNNI2	2.74 x 10^−6^	4.5453

*Note*. Genome‐wide threshold for gene analysis = *p* (.05) /number of genes (18,192) = 2.75 × 10^−6^.

## Discussion

We performed the first GWASes for latent measures of EF in an adolescent sample. The goal of the present study was to further our current understanding of the genetic contributions to specific cognitive measures in adolescence, and the extent of genetic correlations with educationally relevant outcomes.

### SNP Heritability Findings

The highest heritability was found for WM (*h* ^2^_SNP_ = .30), which is similar to other estimates that used well‐replicated tasks (*h* ^2^_SNP_ = _._24–.41; *N*‐back task; Vogler et al., 2014), but higher than tasks with lower validity (*h* ^2^_SNP_ = .05; card memory task; Davies et al., 2016). In line with other complex traits and behaviors, our *h* ^2^_SNP_ estimate is lower than those obtained from twin estimates (*h* ^2^_TWIN_ = .56–1; Friedman et al., 2008). The likely origin of the discrepancy between twin and DNA‐based estimates (often referred to as “still‐missing” heritability) has been extensively debated (e.g., see Manolio et al., 2009), but is likely to be the effects of rare(r) variants not typically captured in a GWAS, along with nonadditive effects. Thus, to consider the full spectrum of genetic influence on WM, further empirical work using very large samples and next generation sequencing approaches will be required.

A more moderate SNP heritability estimate was found for PS (*h* ^2^_SNP_ = .19). Again, as expected this is smaller than twin estimates (*h* ^2^_TWIN_ ∼ 40%–60%; Wright et al., 2001), but larger than that reported in a previous GWAS study (*h* ^2^SNP = .11; Davies et al., 2016). In contrast, common genetic variation failed to account for individual differences in IC (*h* ^2^_SNP_ = −.01). Several explanations could account for this finding. It is possible that individual differences in IC are explained by environmental factors and DNA sequence variants not captured in the GWAS. However, it is also possible that inconsistencies in the parameter settings of the Stop Signal task at age 15 may have complicated the detection of common genetic effects. We attempted to correct for this by regressing out parameter differences from the scores, but this may have resulted in a less reliable measure. Alternatively, our finding could reflect other research showing that IC is not distinguishable from a common EF factor (Friedman & Miyake, 2017; Lee et al., 2013). Although our PCA allowed for components to be correlated (and IC explained the same amount of variance in the cognitive data as WM and PS), the IC component may represent noncognitive or nonheritable variance (Friedman & Miyake, 2017). There has only been one other GWAS (*N* = 12,866) of inhibitory control, and while this study did not estimate SNP heritability it failed to detect any significant genetic associations with the Stroop task (Ibrahim‐Verbaas et al., 2016). Twin study estimates of the Stop Signal task report heritabilities ranging between 26% and 50% (Crosbie et al., 2013; Schachar, Forget‐Dubois, Dionne, Boivin, & Robaey, 2011). Other studies have found moderate twin heritability with little to no SNP heritability in the same sample (Cheesman et al., 2017), with the discrepancy explained by the differences in broad versus narrow sense heritability. It is plausible that non‐cognitive noise present in the individual measures of IC used in twin studies may be contributing to the heritability estimates found (Friedman & Miyake, 2017), or that these estimates are picking up common cognitive variance which is otherwise explained by working memory in this study (Friedman et al., 2008). However, IC has been shown to predict academic achievement in the early years after controlling for other cognitive abilities (Blair & Razza, 2007; Espy et al., 2004). If IC is a distinct cognitive construct, this study suggests that individual differences in adolescence are not explained by common genetic variation. However, our results can also be interpreted as adding to available evidence that IC does not explain unique variance beyond other executive functions.

### GWAS Findings

We failed to identify any robust genetic associations at a genome‐wide threshold with any of the three cognitive latent measures examined. This is not unexpected given the highly polygenic nature of higher‐level cognitive measures and the modest sample size. However, both WM and PS showed a number of suggestive hits (*p* ≤ 1 × 10^–6^) in or near genes that have previously been reported in genetic studies of psychiatric disorders such as schizophrenia and major depression, sleep disorder, and Alzheimer's disease. Interestingly, several of these SNPs have also been associated with cognitive or neurological measures, including performance on the anti‐saccade task and brain volume measures (cingulate and parietal cortex), as well as educational achievement (see Table S3, online Supporting Information). We also found suggestive associations in regions previously associated with subjective well‐being and Parkinson's disease.

### Genetic Correlations

As expected, based on previous findings (e.g., Cragg et al., 2017; Hill et al., 2018; Van der Ven et al., 2012), WM was strongly positively genetically correlated with other cognitive abilities and academic attainment in independent samples. The correlations with intelligence measured in childhood (*r*_G_ = .80 ± .16) and years in education (*r*_G_ = .70 ± .15) were lowest, although the standard errors were large and overlapped with the estimated genetic correlation with lifespan intelligence. These results suggest that genetic variants captured in GWAS studies that affect WM are pleiotropic, and the measures share common underlying biological mechanisms. Further advances in this research will require the identification of genome‐wide significant associations and further dissection (e.g., using Mendelian randomization approaches) to gain insight into the nature and direction of causal relationships between WM, intelligence, *g*, and academic outcomes across development. There was also a significant negative correlation between WM and ADHD (*r*_G_ = −.54), which replicates previous genetic and phenotypic study findings (Martin, Hamshere, Stergiakouli, O'Donovan, & Thapar, 2014; Martinussen, Hayden, Hogg‐Johnson, & Tannock, 2005).

In comparison, the genetic variation in PS in our adolescent sample was found to be more distinct. Genetic correlations with both lifespan and childhood intelligence were half the size of correlations found for WM indicating that genetic causes of individual differences in PS are somewhat independent of those influencing general intelligence and years spent in education (at least when considering the influence of common genetic variants). This is an intriguing result given that PS is thought to be integral to EF and academic achievement (Dodonova & Dodonov, 2012; Gordon, Smith‐Spark, Newton, & Henry, 2018; Kail, 2000). Given that our measure of PS was heritable, it suggests that distinct biological pathways contribute to individual differences in PS compared to those influencing general cognitive function and educational attainment. The correlation with ADHD is moderate, reflecting behavioral research (Walg, Hapfelmeier, El‐Wahsch, & Prior, 2017).

### Gene‐Based Findings

Gene‐based analyses potentially have greater statistical power to detect causal loci because they aggregate correlated SNP effects across a gene, and reduce the multiple testing burden (Kang, Jiang, & Cui, 2013). A gene‐level significant association between WM and Fam181b (Homo sapiens family with sequence similarity 181 member B) was found. Fam181b codes for an intracellular protein and is widely and almost exclusively expressed in the brain, showing enrichment in the caudate, cerebellum, cortex, hippocampus, and hypothalamus (https://gtexportal.org/home/), and has been associated with Alzheimer's disease (Herold et al., 2016). In mice, Fam181b transcripts have been detected early in embryonic brain development (Marks et al., 2016).

A gene‐based association was also found between PS and *TNNI2*. *TNNI2* (Homo sapiens troponin I2, fast skeletal type), is part of a collection of genes involved in governing muscle function, and mutations in this gene are associated with digestive system disease (Jostins et al., 2012) and muscle contractures (Sung et al., 2003; Toydemir & Bamshad, 2009). Speculatively, there could be a plausible physiological relationship between muscle function and physical speed of response, which would not necessarily reflect individual differences in cognition.

## Conclusion

In conclusion, although individual and common EF components and PS have been found to be closely related (Gordon et al., 2018), the contribution of common genetic variation to individual differences in working memory, inhibitory control, and processing speed were found to differ. No SNPs were significantly associated with any of the cognitive measures, but two genes were found to be associated with WM and PS, respectively. Thirty percent of the variance in WM was explained by common SNPs. The same genetic variation also contributes to individual differences in intelligence. SNP heritability for variance in PS was 19%, with approximately 50% of this heritability shared with intelligence. Inhibitory control in contrast appeared to have very low SNP heritability. This might reflect the measure failing to explain (heritable) cognitive variance in the sample, or that individual differences in performance on IC tasks are largely environmentally driven.

## Supporting information


**Appendix S1**. online Supporting Information
