# Supplementary material for: Genome‐Wide Association Study of Latent Cognitive Measures in Adolescence: Genetic Overlap With Intelligence and Education
Source: Mind Brain Educ. 2019 Jun 30;13(3):224–33. doi: 10.1111/mbe.12198 (PMC6771723; doi:10.1111/mbe.12198)
Supplement: Supplementary file 1 — Appendix S1. online Supporting Information [file MBE-13-224-s001.docx]

Supplementary Materials:

**Description of study cohort numbers**

Pregnant women resident in Avon, UK with expected dates of delivery 1st April 1991 to 31st December 1992 were invited to take part in the study. The initial number of pregnancies enrolled is 14,541 (for these at least one questionnaire has been returned or a “Children in Focus” clinic had been attended by 19/07/99). Of these initial pregnancies, there was a total of 14,676 foetuses, resulting in 14,062 live births and 13,988 children who were alive at 1 year of age.

When the oldest children were approximately 7 years of age, an attempt was made to bolster the initial sample with eligible cases who had failed to join the study originally. As a result, when considering variables collected from the age of seven onwards (and potentially abstracted from obstetric notes) there are data available for more than the 14,541 pregnancies mentioned above.

The number of **new pregnancies** not in the initial sample (known as Phase I enrolment) that are currently represented on the built files and reflecting enrolment status at the age of 18 is 706 (452 and 254 recruited during Phases II and III respectively), resulting in an additional 713 children being enrolled. The phases of enrolment are described in more detail in the cohort profile paper (Boyd et al., 2013).

The total sample size for analyses using any data collected after the age of seven is therefore 15,247 pregnancies, resulting in 15,458 foetuses. Of this **total sample** of 15,458 foetuses, 14,775 were **live births** and 14,701 were **alive at 1 year of age**.

A 10% sample of the ALSPAC cohort, known as the **Children in Focus (CiF) group**, attended clinics at the University of Bristol at various time intervals between 4 to 61 months of age. The CiF group were chosen at random from the last 6 months of ALSPAC births (1432 families attended at least one clinic). Excluded were those mothers who had moved out of the area or were lost to follow-up, and those partaking in another study of infant development in Avon.

The study website contains details of all the data that is available through a fully searchable data dictionary (http://www.bris.ac.uk/alspac/researchers/data-access/data-dictionary/).

**Supplementary Table 1:** Component loadings in the cognitive measures principal component analysis. PC: principal component; RT: reaction times.

| *Variable description* | *PC1*  *Slow Processing* | *PC2*  *Working Memory* | *PC3*  *Inhibitory Control* |
| --- | --- | --- | --- |
| Digit vigilance RT (age 13)  Choice RT (age 13)  Simple RT (age 13)  Stop Signal Go trials RT (age 10)  Sky Search Selective attention speed (age 11) | **0.80**  **0.74**  **0.66**  **0.49**  **0.41** | 0.20  −0.13  −0.20  0.01  −0.11 | −0.07  0.07  0.04  0.18  −0.09 |
| 2-back accuracy (age 17)  Digit Vigilance d-prime (age 13)  Counting Span score (age 10)  RT (3-back – 2-back)/(2-back) (age 17)  Dual task decrement score (age 11) | −0.08  0.12  −0.12  0.00  −0.09 | **0.75**  **0.72**  **0.60**  **0.55**  −**0.42** | 0.00  0.01  0.04  0.01  0.04 |
| Stop Signal number of correct Stop trials (age 15)  Stop Signal Go trials RT (age 15)  Stop Signal number of correct Go trials (age 15) | −0.11  0.17  0.02 | 0.14  −0.04  0.18 | **0.83**  **0.79**  −**0.66** |
| Eigenvalues  % of variance explained  α | 2.16  17%  0.65 | 2.11  16%  0.6 | 1.82  14%  0.66 |

**Supplementary Table 2:** Summary of quality control procedures for genome-wide association analysis. ALSPAC: Avon Longitudinal Study of Parents and Children; GWAS: genome-wide association study, LD: linkage disequilibrium, QC: quality control, SNP: single nucleotide polymorphism.

| Procedure | Performed by | Details |
| --- | --- | --- |
| Pre-Imputation sample QC | ALSPAC | ALSPAC children were excluded on the basis of gender mismatches; minimal or excessive heterozygosity; disproportionate levels of individual missingness (>3%) and insufficient sample replication (Identity By Descent < 0.8). Population stratification was assessed by multidimensional scaling analysis and compared with Hapmap II (release 22) European descent (CEU), Han Chinese, Japanese and Yoruba reference populations; all individuals with non-European ancestry were removed and then after combining with maternal genotypes data any with potential ID mismatches were also removed. Cryptic relatedness was measured as proportion of identity by descent (IBD > 0.1). Related subjects that passed all other quality control thresholds were retained during subsequent phasing and imputation leaving a final sample of 8,941 children. |
| Pre-Imputation SNP QC | ALSPAC | SNPs with a minor allele frequency of < 1%, a call rate of < 95% or evidence for violations of Hardy-Weinberg equilibrium (HWE) (p < 5E-7) were removed. A total of 500,527 SNPs passed these quality control filters and these genotypes were then combined with maternal genotypes of which 477,482 matched. A further 11,742 SNPs with genotype missingness above 1%, or that were out of HWE, were removed. |
| Imputation | ALSPAC | Haplotypes were estimated using ShapeIT (v2.r644) (Delaneau, Marchini, & Zagury, 2012) which utilises relatedness during phasing. The phased haplotypes were then imputed to the Haplotype Reference Consortium (HRC) panel of approximately 31,000 phased whole genomes. The HRC panel was phased using ShapeIt v2, and the imputation was performed using Impute V3 (Delaneau et al., 2012). |
| Post-imputation sample QC | Authors | Genetic data were available for 8,941 individuals (4,580 males, 4,361 females). Siblings were removed to leave 8,872 individuals (4,542 males, 4,330 females). Where one sibling had more phenotypic data than the other, that sibling was retained; where both siblings had equal phenotypic data, one sibling was removed at random allowing an equal number of older and younger siblings to be removed.  Ten principal components were created for the 8,872 unrelated individuals in order to control for population structure. Using only observed (i.e., non-imputed) SNPs, short and long-range LD pruning was performed (Price et al., 2008) and then ten principal components were created using the ‘pca’ function in PLINKv1.9 (Purcell et al., 2007). Principal components were plotted to check for outliers that resulted in four participants being removed to leave a final genetic sample of 8,868 individuals. |
| Post-Imputation SNP QC | Authors | Summary statistics for each chromosome were created using QCtool and SNPs were then excluded on the basis of a Minor Allele Frequency < 0.01, with an info score < 0.4, a call rate < 0.95 and HWE of p < 5E-7. 6,319,684 SNPs survived quality control. Following the sample and SNP QC steps described, phenotype and genotype data were retained for a) 4,611 unrelated individuals (2,173 males) for genome-wide analysis of the cognitive data and b) 5,485 individuals (2,602 males) and for the emotion traits. |
| GWAS and post-GWAS QC | Authors | GWAS and post-GWAS quality control was performed by including the 10 principal components in the analysis to account for any population structure. Results were also checked for inflation caused by population structure by interrogating the lambda, value which should be between 0.95 and 1.05. However, as lambda will inflate in the presence of real polygenic signal, it is also necessary to check the LD score intercept, which should be close to 1. Inflation of the LD score intercept represents the presence of population structure and complicates interpretation of results. |

**Supplementary Table 3:** Suggestive single nucleotide polymorphisms (p < 1x10^-6^ ) associated with working memory and processing speed. GWAS: genome-wide association study.

| **Phenotype** | **Chr** | **allele** | **rsid** | **Base position** | **MAF** | **Beta (SE)** | **p-value** | **Gene** | **Previous GWAS hit** |
| --- | --- | --- | --- | --- | --- | --- | --- | --- | --- |
| Working memory | 2 | A:C | rs181853190 | 25900633 | 0.013 | 0.471 (0.11) | 7.2 x 10^-6^ | DTNB* | Schizophrenia, parietal cortex measurement, cingulate cortex measurement |
| Working memory | 2 | A:G | rs7566497 | 183724622 | 0.039 | 0.269 (0.05) | 5.37 x 10^-7^ | FRZB** | Major Depressive disorder, night sleep phenotypes |
| Working memory | 3 | C:T | rs114144395 | 57395019 | 0.012 | -0.483 (0.10) | 1.39 x 10^-6^ | DNAH12** |  |
| Working memory | 3 | A:T | rs838625 | 143191553 | 0.318 | 0.10 (0.02) | 3.20 x 10^-6^ | SLC9A9** | Night sleep phenotypes, cognitive impairment |
| Working memory | 4 | C:T | rs78890674 | 173886129 | 0.012 | -0.570 (0.12) | 3.15 x 10^-6^ | GALNTL6** | Night sleep phenotypes (neuritic plaques) |
| Working memory | 5 | A:G | rs2089199 | 31910627 | 0.390 | -0.098 (0.02) | 8.8 x 10^-6^ | PDZD2** | Anti-saccade error rate in psychotic disorders |
| Working memory | 5 | A:G | rs10079220 | 57453718 | 0.024 | -0.307 (0.07) | 6.7 x 10^-6^ | PGAM1P1* |  |
| Working memory | 5 | A:C | rs10042036 | 57649291 | 0.219 | -0.115 (0.03) | 5.3 x 10^-6^ | PLK2* | Alzheimer’s |
| Working memory | 5 | C:T | rs60258111 | 60079231 | 0.025 | 0.360 (0.08) | 2.9 x 10^-6^ | ELOVL7** | Schizophrenia, parietal cortex measurement, cingulate cortex measurement, educational attainment, anti-saccade error rate in psychotic disorders |
| Working memory | 6 | A:T | rs551980 | 8282533 | 0.497 | -0.102 (0.02) | 1.6 x 10^-6^ | SLC35B3* |  |
| Working memory | 6 | C:T | rs143248626 | 119256466 | 0.034 | -0.275 (0.06) | 9.1 x 10^-6^ | MCM9** |  |
| Working memory | 7 | C:T | rs117555423 | 9133779 | 0.023 | -0.331 (0.07) | 7.8 x 10^-6^ | NXPH1* | Schizophrenia, parietal cortex measurement, cingulate cortex measurement |
| Working memory | 9 | C:G | rs1333039 | 22065657 | 0.412 | -0.094 (0.02) | 9.5 x 10^-6^ | CDKN2B-AS1** |  |
| Working memory | 11 | A:G | rs474357 | 82437118 | 0.216 | 0.127 (0.03) | 5.9 x 10^-7^ | FAM181B* | Cerebral amyloid deposition in APOEe4 non-carriers |
| Working memory | 11 | A:G | rs523867 | 82479873 | 0.160 | -0.132 (0.03) | 3.4 x 10^-6^ | FAM181B* |  |
| Working memory | 12 | A:G | rs10744264 | 126967956 | 0.105 | -0.164 (0.04) | 5.0 x 10^-6^ | RP5-944M2.3* |  |
| Working memory | 12 | A:C | rs10773290 | 126985979 | 0.172 | -0.135 (0.03) | 2.6 x 10^-6^ | NDUFA5P6* |  |
| Working memory | 14 | A:G | rs28576539 | 96306550 | 0.107 | -0.156 (0.03) | 2.8 x 10^-6^ | LINC00617* |  |
| Working memory | 15 | C:G | rs75024542 | 84192118 | 0.033 | -0.308 (0.06) | 1.3 x 10^-6^ | SH3GL3** | Schizophrenia, height |
| Working memory | 18 | A:G | rs652730 | 77162525 | 0.096 | -0.163 (0.04) | 4.5 x 10^-6^ | NFATC1** |  |
| Working memory | 21 | A:G | rs4816642 | 41110282 | 0.210 | -0.133 (0.03) | 3.3 x 10^-6^ | IGSF5* | Suicide risk |
| Processing Speed | 1 | A:G | rs79914264 | 29685027 | 0.012 | -0.479 (0.11) | 6.9 x 10^-6^ | RP3-437I16.1* |  |
| Processing Speed | 1 | A:G | rs80032087 | 68548953 | 0.051 | 0.219 (0.05) | 8.7 x 10^-6^ | GNG12-AS1* |  |
| Processing Speed | 2 | A:G | rs117204046 | 134023319 | 0.046 | -0.236 (0.05) | 3.7 x 10^-6^ | NCKAP5** | Cognitive decline |
| Processing Speed | 2 | A:C | rs75924665 | 141786613 | 0.110 | 0.165 (0.04) | 1.7 x 10^-6^ | LRP1B** | Educational attainment, night sleep phenotypes, schizophrenia, parietal cortex measurement, cingulate cortex measurement |
| Processing Speed | 4 | A:G | rs77612362 | 180688931 | 0.131 | -0.138 (0.03) | 9.7 x 10^-6^ | SNORD65* |  |
| Processing Speed | 5 | C:T | rs2353010 | 1912480 | 0.255 | 0.118 (0.03) | 1.4 x 10^-6^ | LOC101929081* |  |
| Processing Speed | 5 | A:C | rs61749834 | 11387815 | 0.036 | -0.253 (0.06) | 5.9 x 10^-6^ | CTNND2** | Alzheimer’s, cannabis dependencies, bipolar disorder, schizophrenia |
| Processing Speed | 6 | A:C | rs117328143 | 137654309 | 0.030 | -0.321 (0.07) | 1.2 x 10^-6^ | IFNGR1* | Cognitive decline rate in cognitive impairment, body mass index |
| Processing Speed | 7 | A:G | rs1880318 | 46028167 | 0.208 | -0.122 (0.03) | 2.3 x 10^-6^ | FTLP15* |  |
| Processing Speed | 7 | A:C | rs56957961 | 83255557 | 0.063 | 0.205 (0.04) | 2.6 x 10^-6^ | SEMA3E** |  |
| Processing Speed | 10 | C:T | rs11238581 | 44026005 | 0.221 | 0.119 (0.03) | 3.6 x 10^-6^ | ZNF487* |  |
| Processing Speed | 10 | G:T | rs10999869 | 73292871 | 0.085 | -0.169 (0.04) | 6.7 x 10^-6^ | CDH23** |  |
| Processing Speed | 10 | C:T | rs72812273 | 74872426 | 0.027 | -0.420 (0.09) | 8.1 x 10^-6^ | NUDT13** |  |
| Processing Speed | 12 | C:T | rs58366817 | 47934532 | 0.071 | -0.200 (0.04) | 1.2 x 10^-6^ | RPAP3* |  |
| Processing Speed | 13 | C:T | rs73176740 | 38996158 | 0.023 | -0.361 (0.08) | 9.1 x 10^-6^ | UFM1* LINC00437* | Aggressiveness in attention deficit and hyperactivity disorder, schizophrenia, anti-saccade error rate in psychotic disorders |
| Processing Speed | 13 | A:T | rs72632563 | 88930653 | 0.067 | 0.187 (0.04) | 8.0 x 10^-6^ | RPL29P29* |  |
| Processing Speed | 14 | G:T | rs7144325 | 37038310 | 0.420 | -0.095 (0.02) | 6.7 x 10^-6^ | RPL29P3* Other snps (NKX2-1-AS1**, PHKBP2**, RPL29P3**, NKX2-8**) | Intelligence, cognitive ability, educational attainment, general cognitive ability |
| Processing Speed | 15 | C:T | rs79896452 | 38589654 | 0.037 | 0.260 (0.06) | 4.3 x 10^-6^ | SPRED1** | Psychosis |
| Processing Speed | 15 | A:G | rs117831562 | 38684951 | 0.019 | -0.346 (0.08) | 7.7 x 10^-6^ | SPRED1* |  |
| Processing Speed | 15 | C:T | rs187701342 | 81911145 | 0.057 | -0.229 (0.05) | 1.2 x 10^-6^ | LOC101929655* | Post-traumatic stress disorder, Parkinson’s |
| Processing Speed | 16 | C:T | rs71399907 | 70032959 | 0.021 | 0.377 (0.08) | 1.1 x 10^-6^ | PDXDC2P** |  |
| Processing Speed | 17 | A:G | rs138181598 | 43997525 | 0.013 | -0.463 (0.10) | 8.9 x 10^-6^ | MAPT** | Eduational attainment, Alzheimer’s, cognitive decline rate. |
| Processing Speed | 18 | G:T | rs3752060 | 39145147 | 0.031 | 0.296 (0.06) | 1.4 x 10^-6^ | KC6* |  |
| Processing Speed | 18 | A:G | rs78392342 | 39153288 | 0.017 | 0.367 (0.08) | 7.9 x 10^-6^ | KC6* |  |
| Processing Speed | 18 | C:T | rs117911120 | 61581325 | 0.012 | -0.468 (0.11) | 7.9 x 10^-6^ | SERPINB10** |  |
| Processing Speed | 19 | C:T | rs12982734 | 38593151 | 0.335 | 0.099 (0.02) | 6.4 x 10^-6^ | SIPA1L3** | Subjective well-being, life satisfaction |
| Processing Speed | 22 | A:G | rs228912 | 37503668 | 0.249 | 0.107 (0.02) | 9.9 x 10^-6^ | TMPRSS6** |  |

*nearest gene ** in gene, *Chr* = chromosome, *MAF* = minor allele frequency, rsid= single nucleotide polymorphism accession number, *Previous GWAS* = gene association as reported in the NHGRI-EBI Catalog of published genome-wide association studies (MacArther et al., 2017).

**Supplementary References**

Boyd, A., Golding, J., Macleod, J., Lawlor, D. A., Fraser, A., Henderson, J., … (2013). Cohort profile: The ‘Children of the 90s’; the index offspring of The Avon Longitudinal Study of Parents and Children (ALSPAC). *International Journal of Epidemiology*, *42*(1), 111–127.

Delaneau, O., Marchini, J., & Zagury, J.-F. (2012). A linear complexity phasing method for thousands of genomes. *Nature Methods*, *9*(2), 179–181.

MacArthur J, Bowler E, Cerezo M, Gil L, Hall P, Hastings E, Junkins H, McMahon A, Milano A, Morales J, Pendlington Z, Welter D, Burdett T, Hindorff L, Flicek P, Cunningham F, and Parkinson H (2017). The new NHGRI-EBI Catalog of published genome-wide association studies (GWAS Catalog). *Nucleic Acids Research*, 45 (Database issue): D896-D901.

Price, A. L., Weale, M. E., Patterson, N., Myers, S. R., Need, A. C., Shianna, K. V., … Reich, D. (2008). Long-Range LD Can Confound Genome Scans in Admixed Populations. *American Journal of Human Genetics*, *83*(1), 132–135.

Purcell, S., Neale, B., Todd-Brown, K., Thomas, L., Ferreira, M. A. R., Bender, D., … Sham, P. C. (2007). PLINK: a tool set for whole-genome association and population-based linkage analyses. *American Journal of Human Genetics*, *81*(3), 559–575.
